# Genome-wide association studies for economically important traits in mink using copy number variation

**DOI:** 10.1038/s41598-023-50497-3

**Published:** 2024-01-02

**Authors:** Pourya Davoudi, Duy Ngoc Do, Stefanie Colombo, Bruce Rathgeber, Mehdi Sargolzaei, Graham Plastow, Zhiquan Wang, Guoyu Hu, Shafagh Valipour, Younes Miar

**Affiliations:** 1 Department of Animal Science and Aquaculture, Dalhousie University, Truro, NS Canada; 2 Department of Pathobiology, University of Guelph, Guelph, ON Canada; 3 Select Sires Inc., Plain City, OH USA; 4 Livestock Gentec, Department of Agricultural, Food and Nutritional Science, University of Alberta, Edmonton, AB Canada

**Keywords:** Animal breeding, Quantitative trait, Computational biology and bioinformatics

## Abstract

Copy number variations (CNVs) are structural variants consisting of duplications and deletions of DNA segments, which are known to play important roles in the genetics of complex traits in livestock species. However, CNV-based genome-wide association studies (GWAS) have remained unexplored in American mink. Therefore, the purpose of the current study was to investigate the association between CNVs and complex traits in American mink. A CNV-based GWAS was performed with the ParseCNV2 software program using deregressed estimated breeding values of 27 traits as pseudophenotypes, categorized into traits of growth and feed efficiency, reproduction, pelt quality, and Aleutian disease tests. The study identified a total of 10,137 CNVs (6968 duplications and 3169 deletions) using the Affymetrix Mink 70K single nucleotide polymorphism (SNP) array in 2986 American mink. The association analyses identified 250 CNV regions (CNVRs) associated with at least one of the studied traits. These CNVRs overlapped with a total of 320 potential candidate genes, and among them, several genes have been known to be related to the traits such as *ARID1B*, *APPL1*, *TOX*, and *GPC5* (growth and feed efficiency traits); *GRM1*, *RNASE10*, *WNT3*, *WNT3A*, and *WNT9B* (reproduction traits); *MYO10*, and *LIMS1* (pelt quality traits); and *IFNGR2*, *APEX1*, *UBE3A*, and *STX11* (Aleutian disease tests). Overall, the results of the study provide potential candidate genes that may regulate economically important traits and therefore may be used as genetic markers in mink genomic breeding programs.

## Introduction

American mink (*Neogale vison*) is one of the most important animals in the global fur industry, yet requires highly efficient breeding programs to deal with challenges faced in mink production systems^1^. Several studies have been carried out to discern the genetics of complex traits affecting the sustainability of mink production, including growth^2,3^, feed efficiency^4,5^, disease resistance^6–9^, pelt quality^10–12^, and reproduction traits^13–15^. Understanding the genetic architecture underlying such traits using genome-wide association studies (GWAS) might contribute to expediting the genetic progress through selection, and therefore enhance the production efficiency of the mink industry.

Copy number variations (CNVs) refer to frequently observed structural variations in the form of deletions or duplications greater than 50 base pairs^16^, which cover more of the genome (total bases) and have a higher mutation rate than single nucleotide polymorphisms (SNPs)^17^. Similar to SNPs, CNVs can be applied to detect associations with traits of economic interest in livestock species, and therefore, are considered complementary sources to explain genetic variation contributing to differences in phenotypes^18^. Over the past decade, multiple studies have been carried out to examine the association between CNVs with several important phenotypes in livestock species, such as reproduction^19,20^, health^21–23^, feed efficiency and growth^24–26^, and performance traits^27–29^.

The availability of a high-quality chromosome-based genome assembly^1^ and a genome-wide SNP array for American mink facilitates the identification of genetic variations underlying economically important traits. Recently, Davoudi et al.^4^ characterized the CNVs in American mink using whole-genome sequencing data. However, up to now, the CNV-based GWAS with economically important traits has not been reported in mink. Therefore, this study aimed to identify CNV in a large sample of genotyped mink and perform CNV-based GWAS analyses for Aleutian disease tests, growth and feed efficiency, reproduction and pelt quality traits. In addition, we performed functional annotation of the associated CNV regions (CNVRs) to identify the potential candidate genes for these key traits.

## Materials and methods

All procedures applied for this study were approved by the Dalhousie University Animal Care and Use Committee, and we adhered to the Code of Practice for the Care and Handling of Farmed Mink guidelines^30^ throughout all phases of the research.

### Sampling, DNA extraction, and genotyping

The current study involved individuals from two separate farms: the Canadian Center for Fur Animal Research (CCFAR) at Dalhousie University, Faculty of Agriculture in Truro, Nova Scotia, Canada, comprising 1411 individuals, and Millbank Fur Farm (MFF) in Rockwood, Ontario, Canada, consisting of 1562 individuals. More details about the animals utilized in this study can be found in our previous work by Hu et al.^31^. Mink were raised in standard farming settings, receiving diets from the byproducts of human food production. These diets were adjusted to meet the specific needs of the animals in each production phase. Detailed information about the feed ingredients, chemical composition, and energy content of these diets during different periods can be found in our prior publication^32^.

DNA was extracted from tongue tissue of the animals utilizing the DNeasy Blood and Tissue Kit (Qiagen, Hilden, Germany) following the manufacturer’s instructions. Evaluation of DNA quantity and quality was conducted using a NanoDrop ND-1000 spectrophotometer (NanoDrop Technologies Inc., Wilmington, DE), with consistent 260/280 nm readings falling between 1.7 and 2.0 across all samples. After reaching a final concentration of 500 ng, the samples underwent quality checks for DNA and were subsequently genotyped using the Axiom Affymetrix Mink 70K array (Neogen, Lincoln, Nebraska, United States)^33^.

### CNV detection

Initially, the Axiom™ Analysis Suite (Affymetrix^®^) was applied to perform quality control of raw intensity files and filter genotypes based on dish QC (DQC) values less than 0.82, and a minimum call rate of 97%, following the ‘*Best Genotyping Practices’* Workflow described in Axiom™ Genotyping Solution Data Analysis Guide^34^. The SNPs in sex chromosomes were excluded and only those that passed the quality control were kept for further analyses. The final dataset contained 47,644 SNPs that were located on autosomal chromosomes.

The CNV detection was performed with PennCNV v.1.0.5 software^35^, using the signal intensity ratios (Log R Ratio, LRR) and allelic frequencies (B Allele Frequency, BAF) obtained from the Axiom^®^ CNV Summary Tool software (Affymetrix^®^). Then, the PFB (Population Frequency of B allele) file was compiled based on the BAF of each marker in the whole population, using the PennCNV *‘compile_pfb.pl’* function. The GC content around each SNP marker is known to affect the signal strength through the potential interference of genomic waves^36^. Therefore, we first estimated the percentage of GC content of 1-Mb genomic regions surrounding each marker (500 kb on each side) using faToTwoBit and hgGcPercent tools provided by UCSC Genome Browser^37^ and the FASTA information of the American mink genome assembly^1^. Next, the GC content file was implemented in PennCNV by *‘-gcmodel’* function, which applies a regression model for adjusting the high GC content and recovers samples affected by genomic waves^38^. To achieve high-confidence CNV calls, quality control was applied with the following criteria: standard deviations for LRR < 0.35, BAF drift < 0.01, and waviness factor value between − 0.05 and 0.05. We only retained those CNVs longer than 1 kb in length including at least three consecutive SNPs located on autosomal chromosomes^28^. Finally, 2063 high-quality samples were kept for subsequent analyses.

### Deregressed EBV values

The deregressed estimated breeding values (dEBVs) were calculated for 27 economically important traits, including 11 growth and feed efficiency traits, eight reproduction traits, five pelt quality traits, and three Aleutian disease tests. The growth and feed efficiency traits include harvest weight (HW), harvest length (HL), final body weight (FBW), final body length (FBL), daily feed intake (DFI), average daily gain (ADG), feed conversion ratio (FCR), residual feed intake (RFI), residual gain (RG), residual intake and gain (RIG), and Kleiber ratio (KR). The reproduction traits include gestation length (GL), total number of kits born (TB), number of kits alive at birth (LB), number of kits alive at weaning (LW), survival rate at birth (SB), average kit weight per litter at birth (AWB), average kit weight per litter at weaning (AWW), and survival rate at weaning (SW). The pelt quality traits include dried pelt size (DPS), overall quality of dried pelt (DQU), dried pelt nap size (DNAP), live grading overall quality of fur (LQU), and live grading nap size (LNAP). The Aleutian disease tests include counterimmunoelectrophoresis (CIEP), the Aleutian mink disease virus (AMDV) capsid protein-based enzyme-linked immunosorbent assay (ELISA-P), and the AMDV antigen-based enzyme-linked immunosorbent assay (ELISA-G). Breeding values were estimated for all individuals using different animal models for growth and feed efficiency described in detail by^4^, for reproduction traits described in detail by^13^, for pelt quality traits described in detail by^10^, and for Aleutian disease tests described in detail by^7^.

The EBV reliabilities were calculated using the following formula:

$$EBV \,\,reliabilities =1- \frac{prediction \,\,error \,\,variance}{additive\,\, genetic \,\,variance \,\,of \,\,the \,\,trait}$$

Next, the EBV reliabilities were applied to calculate the dEBVs using the method proposed by^39^. The calculations were performed by the ‘*wideDRP’* function in DRP package^40^ in the R environment^41^, by setting the estimated heritability and the default value of 0.5 for the c parameter, which indicates the proportion of genetic variance not explained by markers. The descriptive statistics of the dEBVs for all traits are summarized in Table 1. We removed the animals with dEBV reliability lower than 0.20. The dEBVs were used as the pseudo-phenotype for the association analyses.

**Table 1:** Descriptive statistics of the deregressed EBV (dEBVs) for growth, feed efficiency, Aleutian disease tests, pelt quality and reproduction traits in American mink.

Trait	Abbreviations	Numbers	Mean	SD	dEBVs	
					Min	Max
Harvest weight	HW	1985	− 0.01	0.30	− 1.82	1.92
Harvest length	HL	1921	0.10	2.03	− 7.92	9.91
Final body weight	FBW	1037	0.00	0.30	− 1.15	1.37
Final body length	FBL	1038	− 0.08	1.98	− 6.59	5.83
Daily feed intake	DFI	1872	0.01	0.07	− 0.15	0.23
Average daily gain	ADG	1044	− 0.02	2.19	− 10.14	9.18
Feed conversion ratio	FCR	1036	0.34	8.60	− 23.39	61.52
Residual feed intake	RFI	1044	0.00	0.02	− 0.12	0.14
Residual gain	RG	1042	− 0.04	1.51	− 7.19	5.70
Residual intake and gain	RIG	1043	− 0.04	1.48	− 6.76	6.43
Kleiber ratio	KR	1044	− 0.01	1.15	− 6.05	4.64
Counterimmunoelectrophoresis test	CIEP	1356	0.00	0.12	− 0.63	0.34
VP2 based enzyme-linked immunosorbent assay test	ELISA-P	1356	0.04	2.12	− 5.16	10.34
AMDV-G based enzyme-linked immunosorbent assay test	ELISA-G	1356	− 0.10	2.23	− 6.39	10.83
Gestation length	GL	1321	− 0.44	2.74	− 29.01	25.43
Total number of kits born	TB	1321	0.25	1.37	− 6.51	11.10
Number of kits alive at birth	LB	1319	0.35	1.26	− 5.15	9.14
Number of kits alive at weaning	LW	1314	− 0.04	1.20	− 8.51	7.39
Survival rate at birth	SB	1169	2.39	7.77	− 61.69	46.51
Average kit weight per litter at birth	AWB	1168	0.01	0.83	− 4.90	5.98
Average kit weight per litter at weaning	AWW	1167	0.95	23.02	− 168.37	132.25
Survival rate at weaning	SW	1166	− 0.27	14.26	− 86.29	51.27
Dried pelt size	DPS	1169	0.02	0.70	− 7.20	8.24
Overall quality of dried pelt	DQU	1148	− 0.01	0.56	− 3.92	2.17
Dried pelt nap size	DNAP	1159	0.17	1.10	− 4.91	4.94
Live grading overall quality of fur	LQU	1260	0.05	0.56	− 3.96	3.79
Live grading nap size	LNAP	1260	0.14	0.79	− 3.84	3.60

### CNV association analysis

ParseCNV2 software^42^, which integrates PLINK^43^ for association analyses, was used to detect the association between CNV and dEBVs of the studied traits. ParseCNV2 software converts the CNV events into probe-based statistics for individual CNVs^42^. Since CNV boundaries differ among individuals, it may be difficult to determine the exact start and end points of CNVs, which makes it challenging to classify different CNVs. Therefore, the CNV association tests were conducted for deletions or duplications separately at the probe level. The following model was applied for association testing:

$${\varvec{y}}={\varvec{X}}{\varvec{b}}+{\varvec{e}},$$

where ${\varvec{y}}$ is the vector of dEBVs, ${\varvec{X}}$ is the design matrix relating dEBVs to fixed effect of one CNV at a time, ${\varvec{b}}$ is the fixed effect of CNV, and ${\varvec{e}}$ is the vector of random residual effects. The association test output was used to merge neighboring SNPs in proximity (less than 1 Mb apart) with comparable association significance (± 1 log p-value) into CNV regions (CNVRs), which constitute a genomic span of at least two consecutive probes. The local lowest P-value for identified probes was chosen to indicate the significant level of the whole CNVRs. To consider multiple testing correction, a threshold less than 5 × 10^−4^ was applied to consider a CNVR significantly associated with the phenotypes, as proposed by the ParseCNV2 developers^42,44^.

### Gene annotation

The list of genes in the latest American mink reference genome (ASM_NN_V1)^1^ was downloaded from the NCBI and the *‘intersect’* function in Bedtools^45^ was used to detect the genes that overlapped with significant CNV regions. Finally, an extensive review of the literature was performed to investigate the biological function of identified candidate genes.

## Results

### CNV identification and distribution

**Figure 1:**
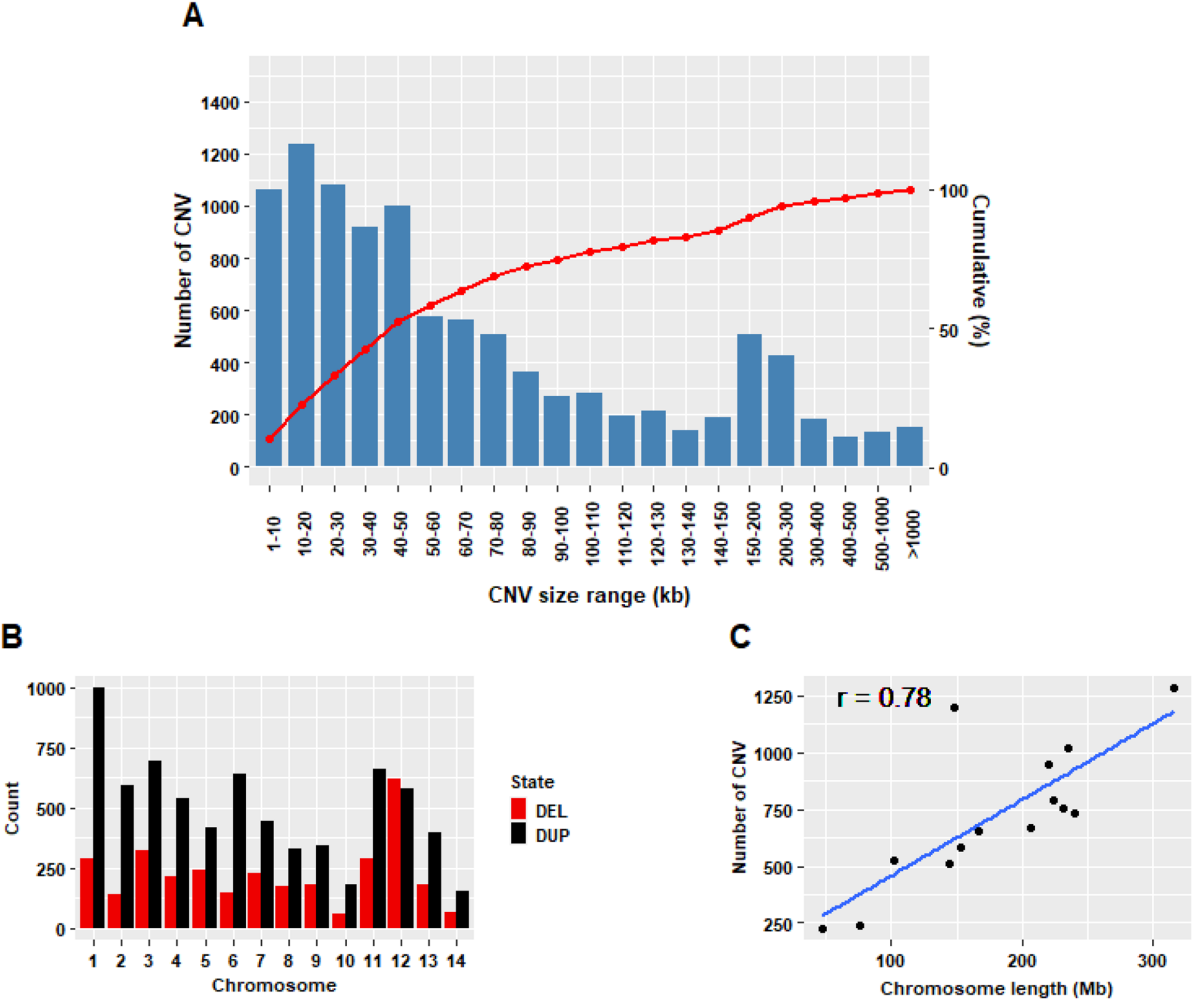
Graphical representation of identified CNVs: (**A**) Distribution of CNV sizes, (**B**) numbers of CNVs identified across autosomal chromosomes, (**C**) correlation between CNV numbers and chromosome length.

Using the PennCNV software based on the Hidden Markov Model method^35^, a total of 10,137 CNV events were identified from 2063 individuals that passed the quality control criteria (Supplementary Table S1). While PennCNV is extensively utilized for CNV detection in genotyping array data, it is essential to note its limitations. The internal HMM model applied in the software specifically considers successive SNPs at each step, making it particularly sensitive to local noise. This sensitivity often results in false positives, over-segmentation (where a true CNV is incorrectly divided into smaller segments), and generally imprecise boundaries in the PennCNV calls^46^. Among the total identified CNVs, 6968 (68.74%) were duplications and 3169 (31.26%) were deletions, with a deletions/duplications CNV ratio of 0.45. The length of the CNV events ranged from 1.05 to 6148.34 kb, with an average size of 109.69 kb. Table 2 presents the descriptive statistics of the identified CNVs in the American mink genome. Analysis of the distribution of CNV size showed that approximately half of the CNVs ranged from 1 to 50 kb, with relatively rare CNV events (3.97%) larger than 500 kb (Fig. 1a). The number of CNVs on each chromosome and the chromosome length showed a strong positive linear correlation (Fig. 1c, r = 0.78), such that 1282 CNVs were identified for the largest chromosome (Chromosome 1) and 225 CNVs for the smallest chromosome (chromosome 14; Fig. 1b).

**Table 2:** Descriptive statistics of CNVs detected in American mink genome.

CNV		Number	Mean	Length (bp)	
				Minimum	Maximum
Deletion		3169	98,984.12	1049	2,956,427
Duplication		6968	114,555.32	1128	6,148,335
Overall		10,137	109,687.54	1049	6,148,335

### Association analyses

**Figure 2:**
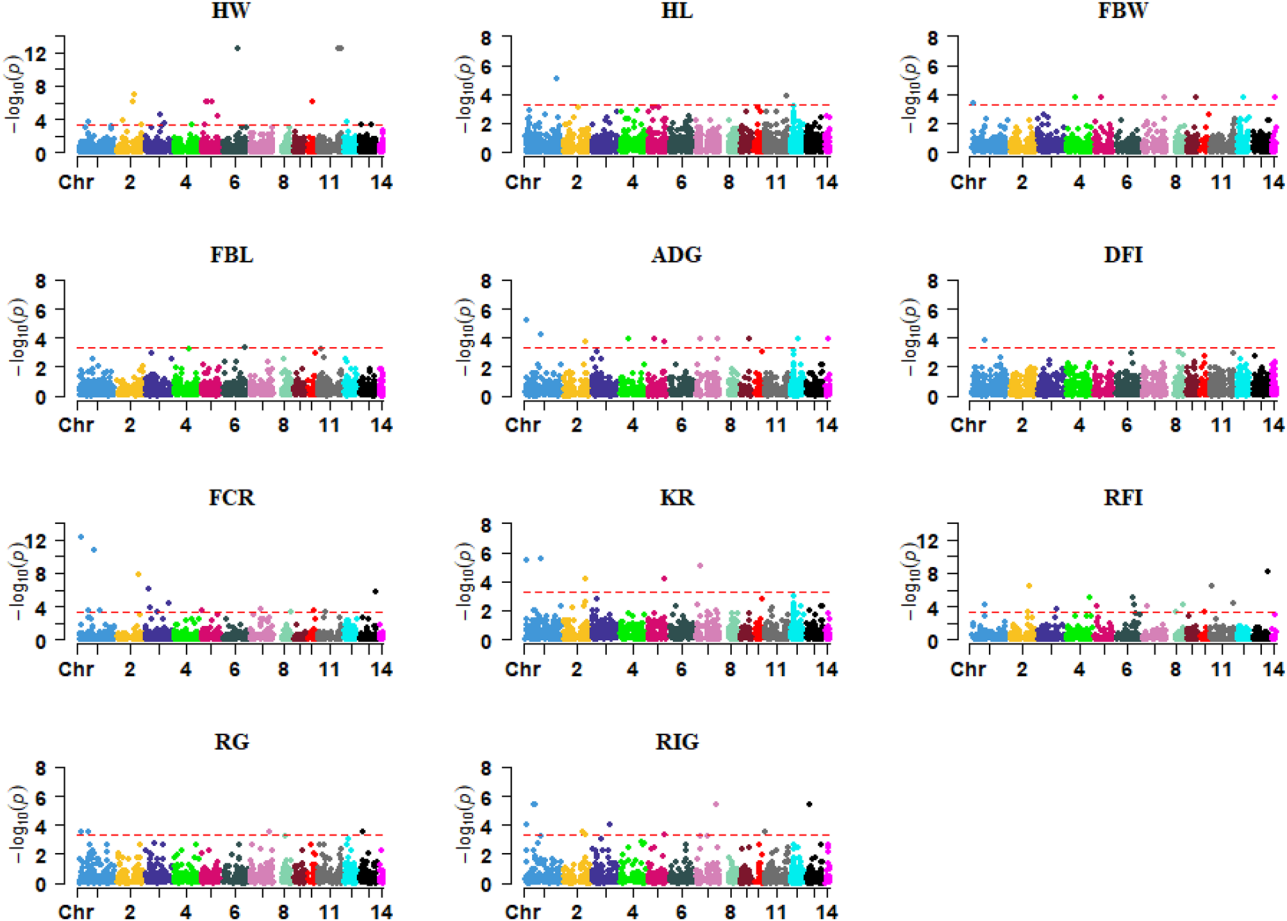
Manhattan plots for CNV regions across the 14 autosomal chromosomes associated with feed efficiency and growth traits. The horizontal line in each plot represents the threshold for significance (P < 0.0005) suggested by ParseCNV2 developers. HW: harvest weight; HL: harvest length; FBW: final body weight; FBL: final body length; ADG: average daily gain; DFI: daily feed intake; FCR: feed conversion ratio; KR: Kleiber ratio; RFI: residual feed intake; RG: residual gain; RIG: residual intake and gain.

**Figure 3:**
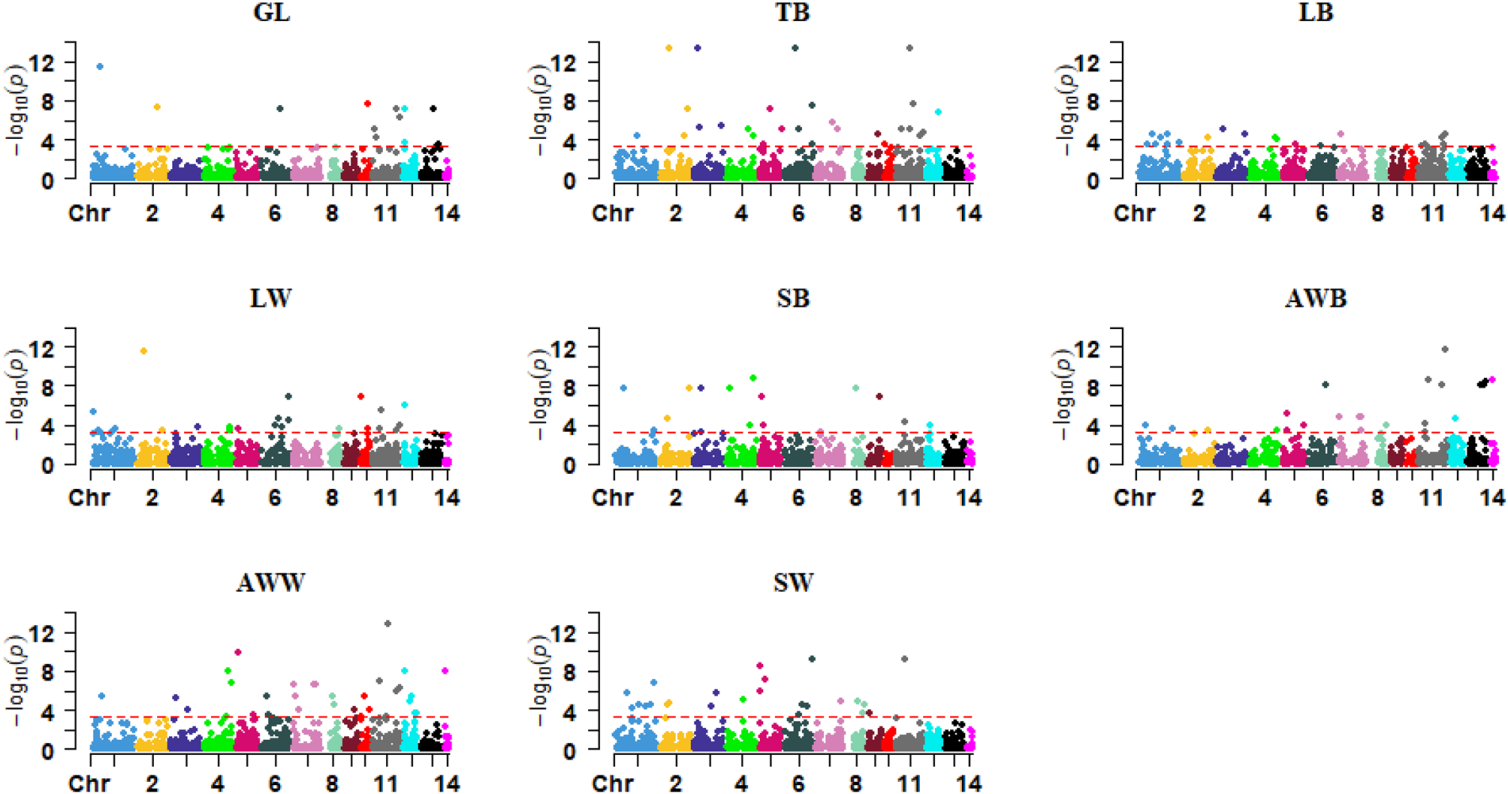
Manhattan plots for CNV regions across the 14 autosomal chromosomes associated with reproduction traits. The horizontal line in each plot represents the threshold for significance ( P < 0.0005) suggested by ParseCNV2 developers. GL: gestation length; TB: total number of kits born; LB: number of kits alive at birth; LW: number of kits alive at weaning; SB: survival rate at birth; AWB: average kit weight per litter at birth; AWW: average kit weight per litter at weaning; SW: survival rate at weaning.

**Figure 4:**
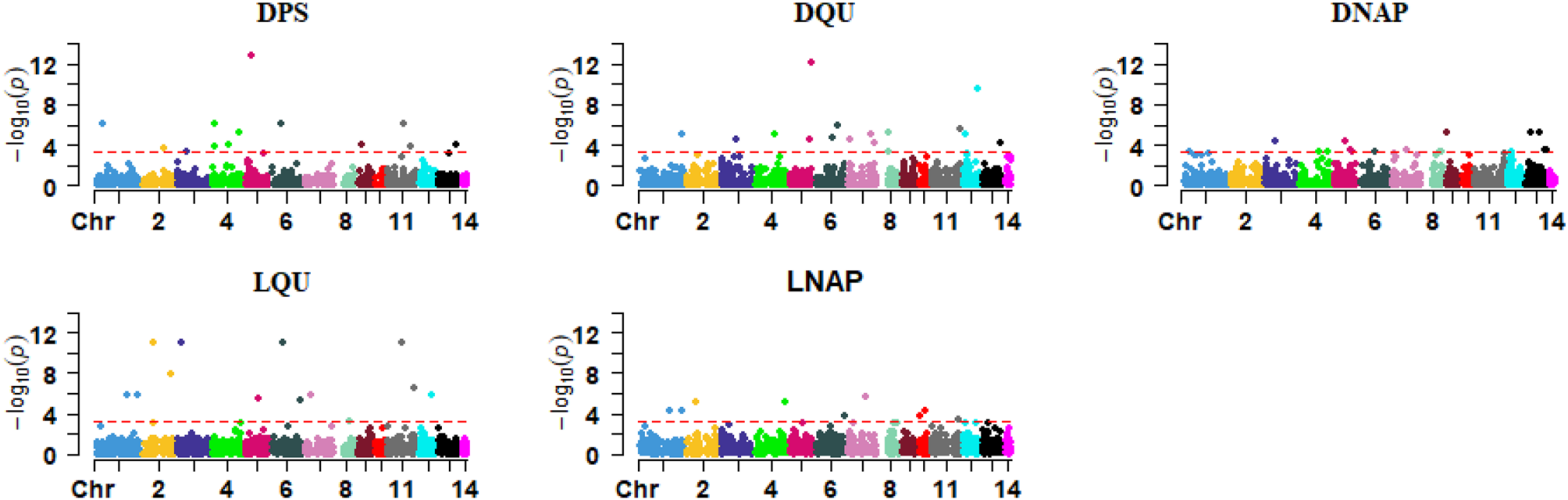
Manhattan plots for CNV regions across the 14 autosomal chromosomes associated with pelt quality traits. The horizontal line in each plot represents the threshold for significance (P < 0.0005) suggested by ParseCNV2 developers. DPS: dried pelt size; DQU: overall quality of dried pelt; DNAP: dried pelt nap size; LQU: live grading overall quality of fur; LNAP: live grading nap size.

**Figure 5:**
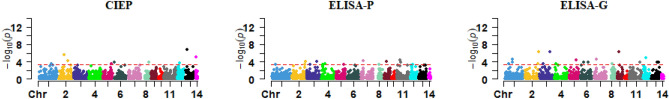
Manhattan plots for CNV regions across the 14 autosomal chromosomes associated with Aleutian disease tests. The horizontal line in each plot represents the threshold for significance (P < 0.0005) suggested by ParseCNV2 developers. CIEP: counterimmunoelectrophoresis test; ELISA-P: VP2 based enzyme-linked immunosorbent assay test; ELISA-G: AMDV-G based enzyme-linked immunosorbent assay test.

In order to explore the effect of CNVs on the complex phenotypes, CNV-based association analyses were carried out for the 27 economically important traits in American mink. Association analyses revealed that 250 CNVRs (71 deletions and 179 duplications) were significantly associated with at least one of the studied traits (P < 0.0005). Manhattan plots for significant CNVRs across the autosomes associated with all studied traits are shown in Figs. 2, 3, 4, 5. These significant regions were identified across all 14 autosomes, while chromosome one showed the largest number (n = 53).

The overview of the top significant CNVRs associated with each studied trait is shown in Table 3. Detailed information for all significant CNVRs with their overlapped candidate genes is provided in Supplementary Table S2. The highest number of significant CNVRs (n = 27) were associated with TB, compromising the most significant region (ID: CNVR54) with a p-value of 3.58 × 10^–14^. In addition, the average length of significant CNVRs was 66.2 kb, ranging from 1.23 to 444.54 kb.

**Table 3:** Overview of the top significant CNVRs associated with all studied traits in American mink.

CNVR ID	Type	Chromosome	Start position	End position	Length (bp)	Associated traits	P-value	Candidate genes
CNVR54	Duplication	2	76,047,394	76,080,498	33,104	TB	3.58E−14	–
CNVR112	Deletion	5	45,720,950	45,756,856	35,906	DPS	1.26E−13	*ITGA3, LOC122907723*
CNVR203	Duplication	11	119,613,197	119,626,953	13,756	AWW	1.44E−13	*LOC122890709, LOC122889431*
CNVR126	Duplication	6	143,324,461	143,377,043	52,582	HW	3.48E−13	–
CNVR1	Duplication	1	10,430,780	10,527,641	96,861	FCR	5.35E−13	*ARID1B*
CNVR113	Deletion	5	158,304,905	158,351,587	46,682	DQU	6.26E−13	*METTL21C, BIVM, CCDC168*
CNVR200	Duplication	11	208,363,505	208,379,126	15,621	AWB	2.24E−12	*DLGAP2*
CNVR56	Duplication	2	51,828,542	51,834,524	5982	LW	2.95E−12	–
CNVR20	Duplication	1	48,882,466	48,894,690	12,224	GL	3.08E−12	*BCKDHB*
CNVR54	Duplication	2	76,047,394	76,080,498	33,104	LQU	8.35E−12	–
CNVR216	Deletion	11	71,374,870	71,389,512	14,642	SW	5.42E−10	*TBC1D9*
CNVR90	Duplication	4	201,592,245	201,635,594	43,349	SB	1.71E−09	*KCND2*
CNVR238	Duplication	13	139,238,384	139,379,378	140,994	RFI	7.32E−09	–
CNVR235	Duplication	13	26,765,710	26,829,160	63,450	CIEP	1.98E−07	–
CNVR40	Duplication	2	237,951,459	238,012,681	61,222	ELISA_G	5.81E−07	–
CNVR159	Deletion	7	130,788,259	130,823,564	35,305	LNAP	1.9E−06	*PIWIL4*
CNVR2	Duplication	1	133,977,608	134,011,851	34,243	KR	2.64E−06	*SYCP2L*
CNVR13	Duplication	1	75,139,496	75,250,132	110,636	RIG	3.97E−06	–
CNVR1	Duplication	1	10,430,780	10,527,641	96,861	ADG	6.29E−06	*ARID1B*
CNVR179	Duplication	9	12,654,424	12,657,997	3573	DNAP	6.66E−06	*TTLL11*
CNVR70	Duplication	3	46,223,063	46,323,843	100,780	LB	7.80E−06	*NDUFA10*
CNVR9	Duplication	1	282,031,840	282,086,441	54,601	HL	9.33E−06	–
CNVR192	Duplication	11	56,914,479	56,922,804	8325	ELISA_P	4.19E−05	*LOC122890225*
CNVR4	Duplication	1	119,299,899	119,337,598	37,699	DFI	1.53E−04	*HSD17B8, SLC39A7, RXRB, COL11A2*
CNVR75	Duplication	4	75,332,844	75,473,471	140,627	FBW	1.81E−04	*TOX*
CNVR12	Duplication	1	11,780,319	11,833,194	52,875	RG	2.84E−04	*NOX3*
CNVR125	Duplication	6	198,790,330	198,804,380	14,050	FBL	4.27E−04	*ASB14, DNAH12, APPL1*

TB: total number of kits born, DPS: dried pelt size, AWW: average kit weight per litter at weaning, HW: harvest weight, FCR: feed conversion ratio, DQU: overall quality of dried pelt, AWB: average kit weight per litter at birth, LW: number of kits alive at weaning, GL: gestation length, LQU: live grading overall quality of fur, SW: survival rate at weaning, SB: survival rate at birth, RFI: residual feed intake, CIEP: counterimmunoelectrophoresis test, ELISA_G: AMDV-G based enzyme-linked immunosorbent assay test, LNAP: Live grading nap size, KR: Kleiber ratio, RIG: residual intake and gain, ADG: average daily gain, DNAP: dried pelt nap size, LB: number of kits alive at birth, HL: harvest length, ELISA_P: VP2 based enzyme-linked immunosorbent assay test, DFI: daily feed intake, FBW: final body weight, RG: residual gain, FBL: final body length.

### Candidate genes within the significant CNVR

We further investigated the candidate genes encompassing the significant CNVRs. The results revealed that a total of 320 potential candidate genes overlapped with significant CNVRs based on the annotation of the American mink genome (Supplementary Table S2). The duplication CNVR on chromosome 7 (ID: CNVR143) overlapped with the highest number of genes (n = 13) while no genes identified within 80 significant CNVRs.

Using the information from the GeneCards database and an extensive literature review, several candidate genes were found to be related to growth and feed efficiency traits (*ARID1B, APPL1, TOX,* and *GPC5*), reproduction traits (*GRM1, RNASE10, WNT3, WNT3A,* and *WNT9B*), pelt quality traits (*MYO10,* and *LIMS1*), and Aleutian disease tests (*IFNGR2, APEX1, UBE3A,* and *STX11*).

## Discussion

Genome-wide association studies using SNP markers have been instrumental in unraveling the underpinning of complex traits^47^. In recent years, CNVs have gained widespread utilization as a supplementary tool in association studies, adding in the identification of genetic variants associated with economically important traits and shedding light on the elucidating the genetic basis of these traits across different livestock species^28,48–51^. To the best of our knowledge, there is no prior research had delved into the realm of CNV associations with diverse phenotypes in American mink.

We conducted the CNV-based association studies using Affymetrix Mink 70K SNP array to identify potential genetic variants associated with dEBVs of 27 different traits such as growth and feed efficiency, reproduction, Aleutian disease tests, and pelt quality traits. In total, 10,137 CNVs were identified, with an average number of five CNVs per sample. Although the average number of detected CNVs per individual is substantially less than our previous study using whole-genome sequencing data (average number of 1647.3), it is in agreement with the results of other studies that used SNP genotyping data with a similar marker density^21,52–54^. It is well-known that the SNP genotyping density affects the number and length of the identified CNVs^21^. The average length of identified CNVs (109.69 kb) is much longer than our previous study with an average size of 7.4 kb, showing differences in resolution and coverage of genome between SNP array and whole genome sequencing data, yet falls within the range of other studies using comparable SNP array datasets^55,56^. A total of 250 significant CNVRs were associated with at least one of the studied traits (P < 0.0005), overlapping with 320 potential candidate genes.

For growth and feed efficiency traits, we identified 86 CNVRs associated with eleven traits. Within these significant CNV segments, we identified *ARID1B, APPL1, TOX,* and *GPC5* genes, which might have large impacts on growth rate and feed efficiency in American mink. The *ARID1B* gene is overlapped with the duplication CNVR1 (Chr1:10,430,780–10,527,641), which was significantly associated with traits such as FBW, ADG, FCR, KR, and RIG. The *ARID1B* gene, which plays a key role in controlling the maturation of neurons during brain development^57^, is the commonly mutated gene in Coffin-Siris syndrome, a genetic disorder characterized by intellectual disability, developmental delay, and growth impairment^58,59^. Yu et al.^60^ reported that the *ARID1B* gene overlapped with identified CNVs in patients with short stature and developmental disorder, indicating the critical function of *ARID1B* mutations in human height regulation. Interestingly, Bovo et al.^61^ detected a region being targeted by selection pressure, harboring the *ARID1B* gene in different pig breeds that grouped by their size, supporting the effect of this gene on body size. The *APPL1* gene located on duplication CNVR125 (Chr6:198,790,330–198,804,380) interacts with several proteins such as adiponectin receptors, AMPK, and Rab5 (a small GTPase downstream of *APPL1*) to regulate apoptosis, cell proliferation, metabolism and insulin sensitivity in energy homeostasis, resulting in increased glucose uptake and fatty acid oxidation^62^. Schweer et al.^63^ reported that the *APPL1* gene is associated with feed efficiency traits in beef cattle through the regulation of glucose.

The *TOX* gene, located in the duplication CNVR75 (Chr4:75,332,844,330–75,473,471), which overlapped with the identified CNVR in our previous study in American mink^64^, was significantly associated with FBW and ADG. The *TOX* gene is a family member of high‐mobility group box proteins and serves as a regulator of gene expression, mostly through modifying the density of the chromatin structure^65^. In cattle, numerous studies demonstrated that the *TOX* gene is associated with feed efficiency^66^, growth^67^, carcass traits^68–70^, and development of puberty^71^. Furthermore, it is shown that the *TOX* gene is associated with weight gain, obesity, and metabolic syndrome-related phenotypes in humans^72^. The *GPC5* gene, which mediates several functions in the control of cell division and growth regulation^73^, is found within a deletion CNVR96 (Chr5:149,166,233–149,199,404), associating with ADG, RIG, and KR. Congruent with these findings, *GPC5* was reported as a candidate gene located within a significant SNP with effects on RIG, RFI, and efficiency of intake in beef cattle^74^. Moreover, other GWAS indicated the association of the *GPC5* gene with human height^75^, body mass index^76^, and body size/body weight in chicken^77,78^.

Regarding the female reproduction traits, we found 168 significant CNVRs, overlapping with several functional genes, among which *GRM1, RNASE10, WNT3, WNT3A,* and *WNT9B* might be the candidate genes related to female reproduction in mink. The *GRM1* gene, which was previously identified in a CNV study in American mink^64^, was located within the duplication CNVR15 (Chr1: 56,870,177–56,925,961), associated with AWB trait. The *GRM1* was a gene of interest reported in several studies to be associated with female reproduction in different livestock species, such as seasonal reproduction in sheep^79^, litter size in goats^80^, number of teats and litter traits in pig^81^, and fertility-related traits in cattle^82^. Interestingly, it was reported that the *GRM1* gene located within structural variations and runs of homozygosity regions associated with litter traits in pigs^83,84^, highlighting the hypothesis that this gene might be a candidate gene for female reproduction in American mink. It was suggested that *RNASE10* gene action in the proximal epididymis is vital for the acquisition of spermatozoa adhesiveness, eventually affecting the mode of sperm transport in the female reproductive tract^85^.

In the current study, several *WNT* family genes were identified to be associated with reproduction traits in American mink, including *WNT3* and *WNT9B* (both found within the duplication CNVR118), and *WNT3A* (overlapped with the deletion CNVR23). It is well-documented that the expression of *WNT3* during the early pregnancy mediates the stromal cell proliferation and trophoblast invasion, eventually affecting the embryonic development^86^. Comparably, the *WNT9B* gene has been reported as one of the key genes associated with inducing the gonadotropin-releasing hormone secretion during follicular development in sheep^87^. Another gene, *WNT3A*, is known as the main regulator of reproductive behavior and follicular activity associated with estrus, which in turn may contribute to the reproductive efficiency in cattle^88^.

The gene annotation within significant CNVRs for pelt quality traits identified some functional candidate genes affecting fur characteristics, such as *MYO10* and *LIMS1*. The *MYO10* gene is an integral member of the myosin family, which is involved in various cellular processes such as dynamic actin remodeling, cell migration and adhesion, and filopodia formation^89,90^. Our findings are in accordance with previous studies indicating that the *MYO10* gene plays a key role in mediating skin pigmentation through regulating melanosome transportation in the skin^91–93^. It was demonstrated that the melanocytes present in the skin control the quantity and types of melanosomes, ultimately determining the coat color^94^. Notably, it was shown that the *MYO10* gene mutation altered the coat color pigmentation pattern in mice, further supporting its role in facilitating melanoblast migration^95^. Interestingly, another member of the myosin superfamily, the *MYO5A* gene, has been widely documented for its impact on coat color phenotype in different species^96–98^, and specifically in American mink^64,99^.

The *LIMS1* gene is located within the duplication CNVR170 (Chr8: 65,501,492–65,735,953), associated with the DQU trait. The *LIMS1* gene involved in the control of cell signaling, adhesion, migration, proliferation, and survival^100^. Several studies demonstrated that the *LIMS1* gene regulates cell adhesion and spreading through the ternary protein complex of integrin-linked kinase (ILK), PINCH, and parvin^101^. To this end, ILK has been reported as a crucial factor for hair morphogenesis^102^. Interestingly, Endo et al.^103^ reported that the *LIMS1* gene was associated with hair morphology and density in East Asians. Furthermore, it was shown that the loss of *LIMS1* gene expression from mouse keratinocytes resulted in impaired hair follicle growth^104^, supporting the importance of this gene on fur development in mink.

Aleutian mink disease virus causes autoimmune disorders in mink by stimulating their immune response to produce antibodies and form immune complexes^8,105^. For CIEP, ELISA-P, and ELISA-G, we identified 9, 12, and 22 significant CNVRs, respectively, which overlapped with several immune-related genes such as *IFNGR2, APEX1, UBE3A,* and *STX11*. The *IFNGR2* gene encodes IFNγR2, which is part of the IFN-γ receptor complex that is overexpressed in an inflammatory environment^106^. It is well established that *IFNGR2* is an important regulator for IFN-γ-STAT1 signaling in T cells^107^, in turn, the dysregulation of the *IFNGR2* gene is associated with a variety of autoimmune diseases^108^. The *APEX1* and *UBE3A* genes overlapped with the duplication CNVR236 (Chr13: 96,258,171–96,346,188) and the deletion CNVR237 (Chr13: 125,264,701–125,304,748), respectively, both associated with ELISA_G, GL and AWB traits. In agreement with our results, Hu et al.^7^ indicated a favorable genetic correlation between Aleutian disease test and reproduction traits in American mink, which suggested the potential for genetic selection of Aleutian disease test traits to alleviate the adverse impact caused by Aleutian disease in mink farms.

The *APEX1* gene (also called *APE1*) encodes a multifunctional protein that regulates the DNA base excision repair and redox activities, the latter demonstrated to be involved in mediating the T helper cell 1 (Th1) response^109^. In addition, it is well-documented that the *APEX1* gene plays a proinflammatory function in stimulating cytokine and chemokine expression, eventually contributing to innate and adaptive immunity processes^110^. It has been confirmed that the *UBE3A* gene, present in both glutamatergic and GABAergic neurons in the brain, functions as a transcriptional regulator of the immune system within the brain^111^. Recently, Zhang et al.^112^ revealed that the *UBE3A* gene within a deletion CNV is associated with the enrichment levels of immune signaling pathways, eventually enhancing antitumor immunity and immunogenicity. The *STX11* gene, which is a member of the SNARE family, is highly expressed in immune tissues such as the thymus, spleen, and lymph nodes, regulating the IFN-γ secretion from natural killer cells, consequently mediating the immune cell function^113,114^.

## Conclusion

For the first time in American mink, the CNV-based GWAS were applied for economically important traits using the Affymetrix Mink 70K SNP array. We identified 10,137 CNVs, including 6968 duplications and 3169 deletions, among which 250 CNVRs were significantly associated with at least one trait. From this, we identified several candidate genes contributing to the growth and feed efficiency (*ARID1B, APPL1, TOX,* and *GPC5*), reproduction (*GRM1, RNASE10, WNT3, WNT3A,* and *WNT9B*), pelt quality (*MYO10,* and *LIMS1*), and Aleutian disease tests (*IFNGR2, APEX1, UBE3A,* and *STX11*). Overall, the associated CNVRs and respective candidate genes in the current study supply additional information, complementary to GWAS analyses solely based on SNP markers, further helping reveal the genetic basis of traits of economic interest in American mink.

### Supplementary Information

Supplementary Table S1.

Supplementary Table S2.

## Data Availability

The datasets used and analyzed during the current study may be available from the corresponding author on academic request.
